# Time series analysis of cutaneous leishmaniasis incidence in Shahroud based on ARIMA model

**DOI:** 10.1186/s12889-023-16121-9

**Published:** 2023-06-20

**Authors:** Mostafa Majidnia, Zahra Ahmadabadi, Poneh Zolfaghari, Ahmad Khosravi

**Affiliations:** 1 Student Research Committee, School of Public Health, Shahroud University of Medical Sciences, Shahroud, Iran; 2 Student Research Committee, School of Medicine, Shahroud University of Medical Sciences, Shahroud, Iran; 3 Vice-chanceller of Health, Shahroud University of Medical Sciences, Shahroud, Iran; 4 Center for Health Related Social and Behavioral Sciences Research, Shahroud University of Medical Sciences, Shahroud, Iran

**Keywords:** ARIMA model, Cutaneous leishmaniasis, Zoonotic disease, Time series analysis

## Abstract

**Background:**

Leishmaniasis is a zoonotic disease and Iran is one of the ten countries with has the highest estimated cases of leishmaniasis. This study aimed to determine the time trend of cutaneous leishmaniasis (CL) incidence using the ARIMA model in Shahroud County, Semnan, Iran.

**Methods:**

In this study, 725 patients with leishmaniasis were selected in the Health Centers of Shahroud during 2009–2020. Demographic characteristics including; history of traveling, history of leishmaniasis, co-morbidity of other family members, history of treatment, underlying disease, and diagnostic measures were collected using the patients’ information listed in the Health Ministry portal. The Box-Jenkins approach was applied to fit the SARIMA model for CL incidence from 2009 to 2020. All statistical analyses were done by using Minitab software version 14.

**Results:**

The mean age of patients was 28.2 ± 21.3 years. The highest and lowest annual incidence of leishmaniasis were in 2018 and 2017, respectively. The average ten-year incidence was 132 per 100,000 population. The highest and lowest incidence of the disease were 592 and 195 for 100,000 population in the years 2011 and 2017, respectively. The best model was SARIMA (3,1,1) (0,1,2)_4_ (AIC: 324.3, BIC: 317.7 and RMSE: 0.167).

**Conclusions:**

This study suggested that time series models would be useful tools for predicting cutaneous leishmaniasis incidence trends; therefore, the SARIMA model could be used in planning public health programs. It will predict the course of the disease in the coming years and run the solutions to reduce the cases of the disease.

**Supplementary Information:**

The online version contains supplementary material available at 10.1186/s12889-023-16121-9.

## Introduction

Leishmaniasis is one of the zoonotic infectious diseases which transmitted to humans and animals. They are classified by the spectrum of clinical manifestations: cutaneous leishmaniasis (CL), visceral (kala-azar), and cutaneous mucosal leishmaniasis [1]. Cutaneous leishmaniasis is the most common form of leishmaniasis, which occurs in both dry (urban) and wet (rural) forms. Leishmania infection is transmitted via the bite of some sandflies. These sandflies are globally distributed and more prevalent in tropical and subtropical countries [1–3]. The incubation period of the disease in rural areas is usually less than four months but the urban type has a longer incubation period and usually lasts approximately two-eight months and even longer [1, 3–5]. World Health Organization (WHO) has identified the disease as one of the six infectious diseases worldwide [6, 7].

In the temperate regions, the activity of sandflies is in the warm seasons of the year and their maximum activities are from June to September. The prevalence and spread of leishmaniasis, in addition to economic, social, and cultural issues, are affected by ecological factors [6, 8, 9]. All the known centers of the disease are between two latitudes of 28 to 42 degrees in the northern latitude. It is the second most important tropical disease after malaria and is endemic in the tropics of the Americas, Africa, and the Indian subcontinent, subtropical regions of southwest Asia, and the Mediterranean region [1, 2, 6, 8, 9]. In Iran, the geographical and climatic conditions are very suitable for the rodents and the reproduction of sandflies mosquitoes that can transmit the disease [1, 2, 6, 8–15].

According to the WHO in 2012, Iran is one of the ten countries with the highest estimated cases of leishmaniasis, which accounts for 70 to 75% of the probable incidence of leishmaniasis in the world. Nearly two million new cases are infected with the disease each year, and between 20,000 and 50,000 people die as a result [7, 16–19].

This study aimed to determine the time trend of cutaneous leishmaniasis using the Autoregressive integrated moving average (ARIMA) model in Shahroud County, Iran from 2009 to 2020. We tried to predict the trend of the disease in the coming years and provide solutions to reduce the cases of the disease.

## Materials and methods

### Study area

Shahroud county is located in the northern margin of Dasht-e Kavir and on the southern slopes of the Alborz mountain range with a geographical position of 36° 25’ latitude, and 54° 58’ longitude with an altitude of 1380 m above sea level in the northeast. The average annual temperature in this county is 14 °C, and the average annual rainfall is 180 mm.

### Data collection

We gathered and classified the monthly data of leishmaniasis incidence from all cities and regions in Shahroud County from January 2009 to December 2020. The information of the study are available on the incidence statistics of infectious diseases of the Data Center of Iran Public Health Science database (http://port.health.gov.ir/mfdc/default.aspx) hosted by the Iran Center for Disease Control and Prevention (Iran CDC).

Data was collected by using a questionnaire, and a linear checklist form which includes demographic characteristics, history of traveling, history of leishmaniasis, comorbidities of another family, treatment history, underlying disease, and diagnostic procedures. In this study, 725 patients with leishmaniasis were identified and treated.

According to the cutaneous leishmaniasis care related guidelines in Iran; it is impossible to conduct the epidemiological study and control of leishmaniasis without the correct diagnosis of the mosquito species, which are the only carriers of leishmaniasis, and play a major role in the spread of the disease. Therefore, the detection networks of sandfly mosquitos’ species, vectors, their abundance determining, and reservoirs identification in the centers of the disease were launched. Based on the latest report on the existed species, and considering the abundance of the dominant species of sandfly mosquito, and the determination of the seeker reservoir, the desired center can be easily identified and planned to fight against the vector and reservoirs [20].

### Statistical analysis

In the time series analysis, through Box-Jenkins methodology, ARIMA and SARIMA models are used to correlate each observation with the previous ones and provide a model to predict the disease [21–24].

First, the Seasonally Correlated Integrated Moving Average (SARIMA) model with Box-Jenkins method was used to determine CL occurrence model. A logarithmic transformation was used for all variables to become stationary input series. Chi-square test was used to detect the seasonal distribution of the disease.

ACF and PACF functions were used to set the ARIMA (p, d, q) and SARIMA (P, D, Q, S) models. Parameter P (Autoregressive) is specified by the PACF diagram and shows the significant delays in the fixed series. Q (Moving Average) in ACF diagram is used to determine the seasonal trend. In the ACF diagram, the seasonal trend is shown by the sinus wave. For the controlling seasonal trend, use the division difference (D = 1). The number of differentials to control the seasonal effect is indicated by D (Differential) and represented by seasonal data (D). Because the data were collected quarterly, S was equal to 4.

The normality of model residues and their independence was used to evaluate the model. If the model can be calculated correctly, the remainder will be natural and independent. Then, the model can be estimated with determining the appropriate and realistic values and plotting them. In this study, Akaike’s information criteria (AIC) and Bayesian information criteria (BIC) were also applied to select the best model. In the analysis, the statistically significant level was set at 0.05. Minitab software version 14 has been performed for model analysis and forecasting.

## Results

### Descriptive statistics

In this study, 725 patients with leishmaniasis were identified and treated from 2009 to 2020 in the Health Centers of Shahroud County. The mean age was 28.2 ± 21.3 years and the highest incidence of the disease was in the age group 41–60 years. During these years, 396 (54.6%) and 329 (45.4%) patients were males and females, respectively. 146 (20.1%) patients had a history of leishmaniasis. The most common site inflicted by lesions was the hip and leg (24%). The average ten-year incidence was 132 per 100,000 population.

Table 1 showed a distribution of leishmaniasis according to the type, history of traveling, and form of lesion. According to the table, the most common leishmaniasis type was major (342, 47.2%). The wet form of lesion was the most distribution of leishmanias during the study period (397, 54.8%).

**Table 1:** Distribution of leishmaniasis according to the type, history of traveling and form of lesion (2009–2020)

Frequency	Percent		
Type of leishmaniasis	*Tropica*	185	25.5
	*Major*	342	47.2
	Unknown	198	27.3
History of traveling to endemic regions	Traveling	210	29
	No traveling	371	51.2
	Not reported	144	19.8
Form of lesion	Dry	175	24.1
	Wet	397	54.8
	Unknown	153	21.1

The highest and lowest incidence were 592 and 195 for 100,000 population in the years 2011 and 2017, respectively. The incidence of leishmaniasis by the year and regions was presented in Table 2.

**Table 2:** Incidence of leishmaniasis in 100 000 inhabitants of Shahroud city during 2009–2020

Region Year	Shahroud	Bastam	Miami	Biarjmand
2009	18.2	48.1	75.3	63.5
2010	6	0	55	8
2011	0	0	545.5	38.6
2012	2	0	144	90
2013	0	0	194	27.7
2014	0	0	253	13.9
2015	0.7	0	186.8	27.7
2016	0	0	168	28.6
2017	0	0	164.3	30.4
2018	0	0	172.5	45.6
2019	0	0	159.2	38.3
2020	0	0	167.3	41.9

### Modeling

For the time series forecasting, the Box-Cox test (theta: -0.19) was used for variance stationary and Dicky-Fuller test (P < 0.05) was used for mean stationary. Due to the instability of the data in terms of variance, the original data was changed to ln to become static (theta: 1.07). By drawing ac and pac and seasonal diagrams, the values ​​(p, d, q) (P, D, Q) were reported (Supplementary Fig. 1). To control the seasonal trend, seasonal differentiation was taken from the data. The result of the chi-square test showed that the disease has a seasonal distribution (χ2 = 25.6, P = 0.007). Also, the geographical distribution of the disease by cities and years from 2009 to 2020 was illustrated seperately (Supplementary Fig. 2).

Finally, two models of SARIMA (3,1,1) (0,1,2)_4_ and SARIMA (3,1,2) (0,1,1)_4_ were selected as the best models and were compared in terms of coefficients and fitting parameters in Table 3. After comparing different SARIMA models by using the likelihood ratio test, the best model was SARIMA (3,1,1) (0,1,2)_4_ (AIC: 324.3, BIC: 317.7 and RMSE: 0.167). All coefficients were statistically significant in the model ARIMA (3,1,1) SARIMA (0,1,2) 4 (P < 0.05) (Table 4).

**Table 3:** Comparison between two final seasonal models for predicting number of cases of leishmaniasis in Shahroud

	Models	R^2^	Ljung-Box test	AIC	BIC	RMSE
		stst	P			
SARIMA (3,1,1) (0,1,2)_4_	0.98	31.05	0.153	324.3	317.7	0.167
SARIMA (3,1,2) (0,1,1)_4_	0.81	32.92	0.081	329.6	341.2	0.249
SARIMA (3,2,1) (0,1,2)_4_	0.68	35.44	0.053	348.1	344.0	0.268
SARIMA (3,2,2) (0,2,2)_4_	0.70	39.02	0.066	352.3	349.9	0.299

**Table 4:** Coefficients and statistics of variables in SARIMA (3,1,1) (0,1,2)_4_ model for predicting number of cases of leishmaniasis in Shahroud

Variables	Coefficients	Standard Error (SE)	CI 95%	Z	P-value*
AR1	-1.019	0.040	-1.097 -0.940	-25.47	0.000
AR2	-1.017	0.040	-1.096 -0.939	-25.37	0.000
AR3	-0.995	0.013	-1.021 -0.969	-74.08	0.000
MA1	0.285	0.145	0.110–0.570	1.97	0.049
MA seasonal	-0.803	0.265	-1.324 -1.281	-3.02	0.003
Constan	-0.333	0.365	-1.050 -0.501	-0.91	0.013
Sigma	12.369	1.873	8.696–16.042	6.6	0.000

*P < 0.05

**Fig. 1:**
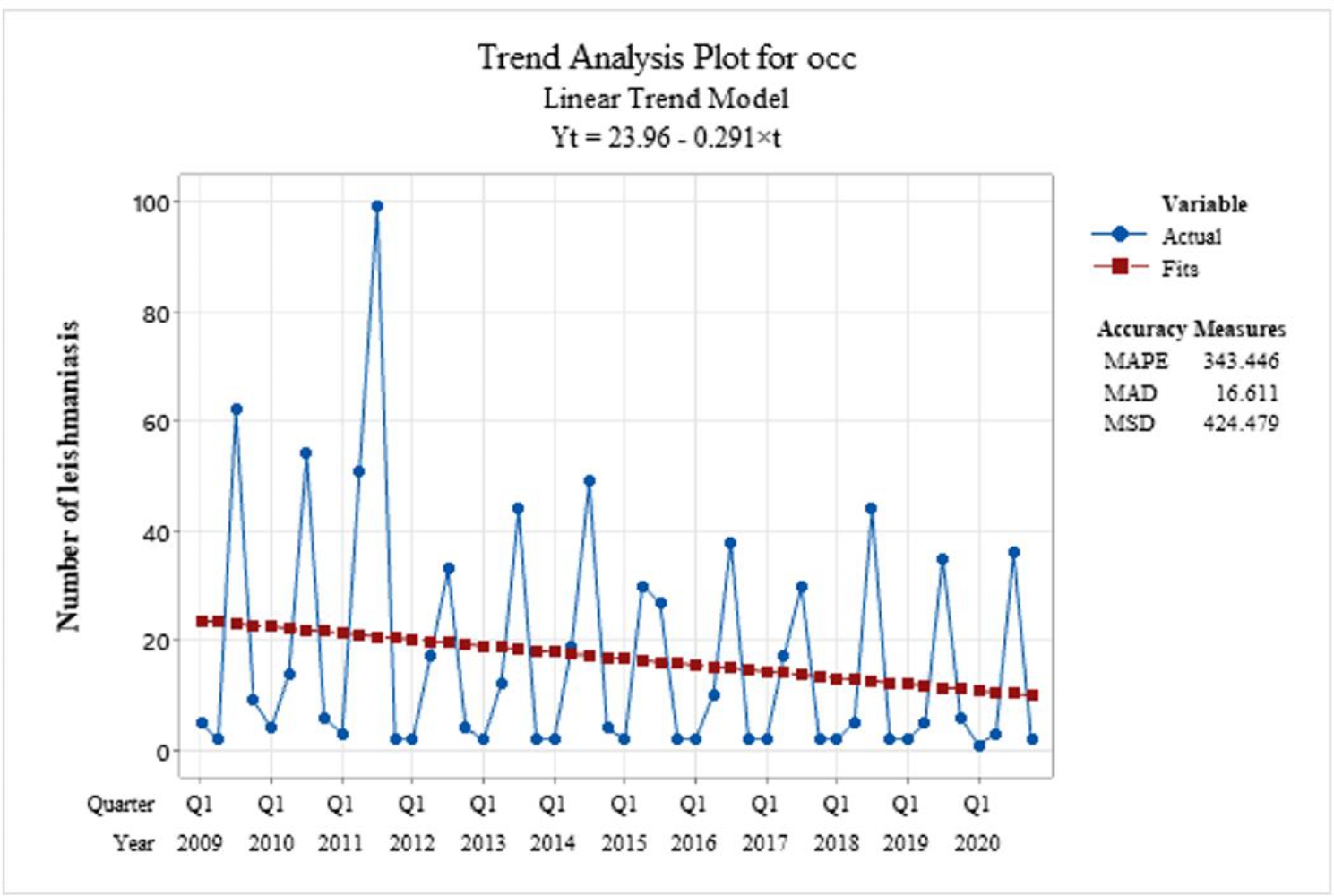
Linear analysis of leishmaniasis seasonally during the study period (2009–2020)

**Fig. 2:**
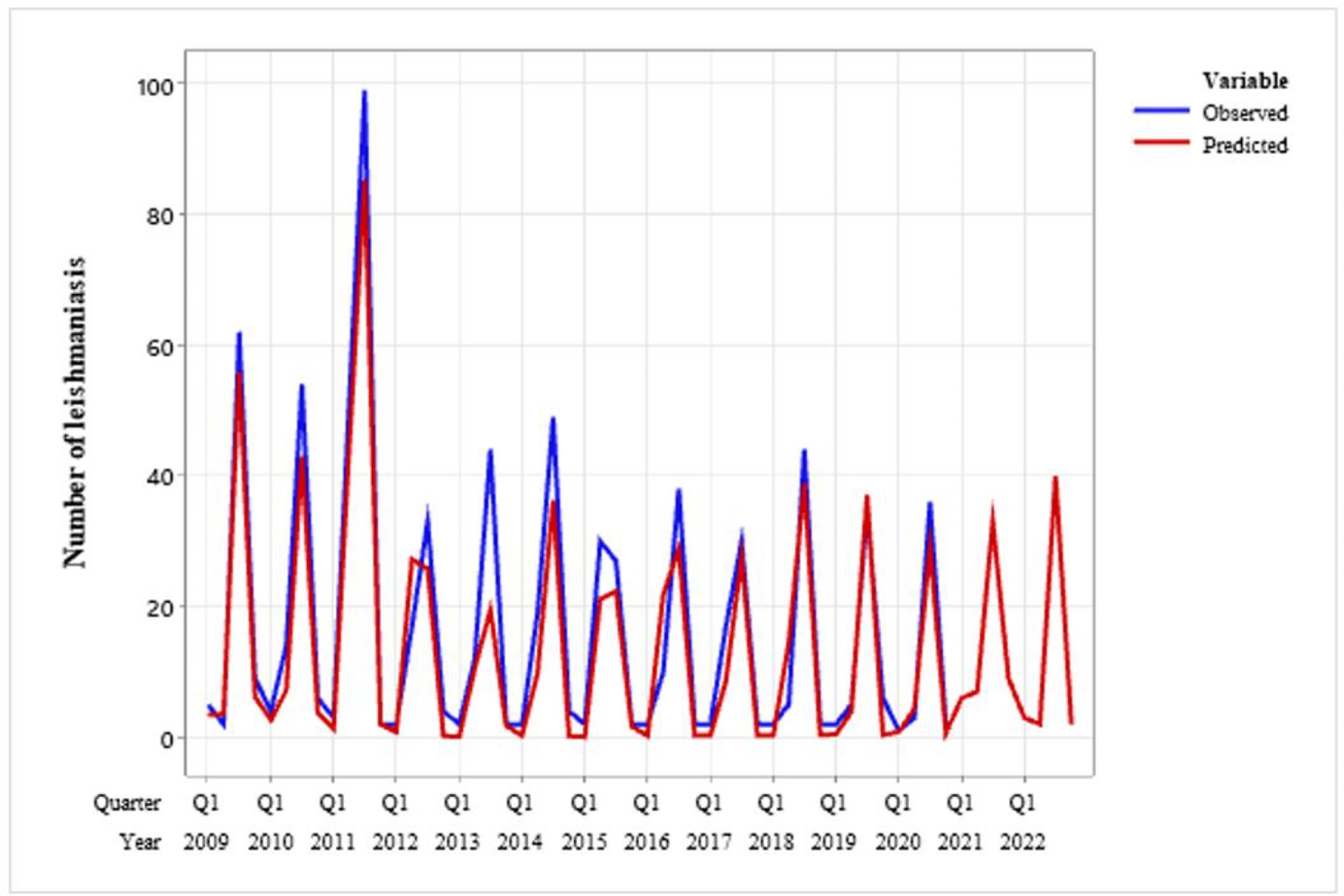
Occurrence trend of leishmaniasis and predicting trend using seasonal model (2009–2022)

Also the trend of the disease analysis was downward trend (Fig. 1). The linear trend equation of leishmaniasis is estimated as Yt = 21.68 − 0.268 × t. According to the model, the trend of the disease is decreased (Fig. 2).

## Discussion

The results showed that Miami had the highest incidence of leishmaniasis from 2009 to 2020 and the best model was ARIMA (3,1,1) SARIMA (0,1,2)_4_. Also, both sexes were susceptible to the disease and the highest incidence was observed in men. Jorjani and his colleagues (2019) found that men had the higher number of CL cases than women. This result was the same as the result of our study [25]. Also Sofizadeh (2016) and his colleagues reached the same result which is consistent with our study [26]. Men constitute the majority of seasonal migrants as workers who wear less clothing and have more environmental and social interactions than women.

The study population included all age groups, but the highest incidence was observed at the ages 41–60 years. In addition, the incidence of leishmaniasis increased in summer and autumn and decreased in winter and spring. This is probably due to the greater activity of sandflies in the spring and summer as the latency of the disease. This can be a result of suitable situation for reproductive of sandflies including degree of 23–28 centigrade and moisture of 70–100% that are commonly occurred in the last months of summer and early autumn [27]. As the same as our result, in Karami study (2013) in Isfahan, the majority of patients were infected in fall. Also most of the patients were in the age group 20–31 years [28]. This result was in contrast with our research which may be related with some factors like the occupation, education, and activity. It can be concluded that the rate of infection in different age groups depend on the study location. Although the incidence of the disease had increased at the beginning of the study (2009) and after 2015 had a steady trend.

The result of the Sharafi study (2017) in the south of Fars showed that SARIMA (4,1,4) (0,1,0)_12_ model in general and SARIMA (4,1,4) (0,1,1)_12_ model at the lower and higher ages than 15 years old could adequately predict the course of the disease in the study area [18]. In the Nikonahad study (2017) in western Iran, SARIMA (1,0,1) (1,0,0)_12_ model was used for the incidence trend of leishmaniasis [17]. According to the Morrone (2011), the best model for predicting was ARIMA (2,0,3). This model had been selected based on observations and the correlation elimination in the residue as detected by the Ljung-Box test and the automatic residual series correlation and partial correlation functions [12]. The result of Kalim Ullah et al. (2016) time series study in Pakistan with the ARIMA method showed that the best ARIMA models were ARIMA (1,0,0) (0,1,0)_12_ and ARIMA (0,0,0) (0,1,0)_12_ with a seasonal period of 12 months, respectively [29]. All studies had shown variables such as air temperature, humidity, rainfall, and altitude had been included in the model except incidence of the disease, which had caused differences in these models with our study. Also in this study, the seasonal trend of the disease had been shown.

The relationship between cutaneous leishmaniasis and vegetation index, rainfall, temperature, humidity, wind, and temperature inversion have been shown in different studies. So, providing humid and warm conditions for carriers can increase the number of infected cases after wet seasons [18, 30–33]. In these areas, continued drought and reduced rainfall, which affects environmental variables, could effect on a declining disease trend. Also it should be noted that each region has a climatic condition, host and carrier of the disease, and the trend of the disease can change based on these conditions. Therefore, assessment of the disease trend should be specific according to the conditions of each region [18].

The declining trend of the disease in this region could be effected on the implementation of controlling programs, including fight against reservoirs (rodents) and vectors and enhance the environment in all the areas. In Iran, many studies had been performed on the effect of rodent programs with P_2_ZN_3_ toxin in reducing the incidence of leishmaniasis; Thus, the implementation of programs that lead to the reduction of disease reservoirs can ultimately reduce the incidence of disease [34, 35].

Impregnation of mosquitoes with permethrin toxin can also disrupt the human transmission chain, thereby reduce the number of vectors. In addition, increasing the level of knowledge about the disease and methods of prevention, which are regularly implemented by the health centers, can help to control the trend of the disease. These issues have been proven in various studies. Also it can be related to the drafting process and the reduction of vegetation due to the effect of environmental conditions, especially climate, on the frequency of carriers and reservoirs of the disease [34–36].

Due to the high rate of leishmaniasis in this region, especially in the city of Miami, timely identification of new cases of leishmaniasis, especially major and wet type, and the start of treatment after a definitive diagnosis requires more attention. Also, the diagnosis should be seriously followed up in rural farmers and ranchers, and try to control, and treat them definitively, and appropriately. Educational sessions and the distribution of pamphlets, tracts in the city and especially in the villages are necessary to increase the level of knowledge among households about leishmaniasis. A workshop or seminar should be held for specialized and general practitioners to retrain and increase their sensitivity to leishmaniasis. It is also suggested that in future studies, other effective factors in increasing the incidence of leishmaniasis, such as stagnant water conditions, public health, as well as medical facilities in this area could be investigated.

The most important limitations of the present study are related to the registration system in which some unreported, undiagnosed, and incorrectly diagnosed cases are eliminated. Some patients with leishmaniasis went to private clinics or self-medication and were not registered in the health centers. To improve the accuracy of this data, active monitoring is needed to detect leishmaniasis cases.

## Conclusions

The results of this study showed that time series models can be useful tools for predicting the incidence of the disease. Predicting disease trends, as an important issue in the rapid alert system, plays an essential role in preventing the spread of disease, providing facilities and controlling diseases. In seasonal diseases, due to the removal of the seasonal component, the SARIMA model can be used to predict the disease trend properly. Therefore, they can be used in planning public health programs and resource management. Meteorological variables such as humidity, rainfall, rainy days, and socio-economic conditions in the models can improve their accuracy.

## Electronic supplementary material

Below is the link to the electronic supplementary material.

Supplementary Material 1

## Data Availability

All data generated and analyzed during this study are included in this article.
