# Supplementary material for: Time series analysis of cutaneous leishmaniasis incidence in Shahroud based on ARIMA model
Source: BMC Public Health. 2023 Jun 20;23:1190. doi: 10.1186/s12889-023-16121-9 (PMC10283195; doi:10.1186/s12889-023-16121-9)

**Supplementary figure 1: Autocorrelation and partial autocorrelation functions calculated using the seasonal differenced, number of CL cases from 2009 to 2020 in Shahroud, Iran**


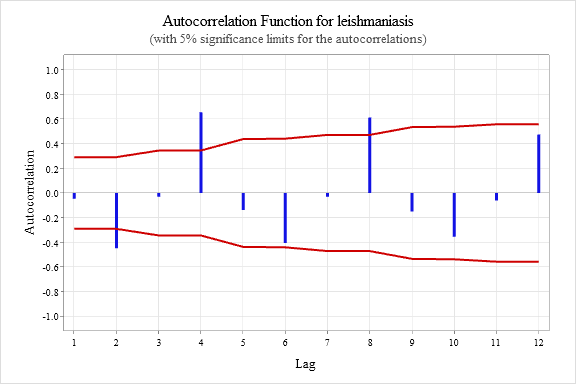


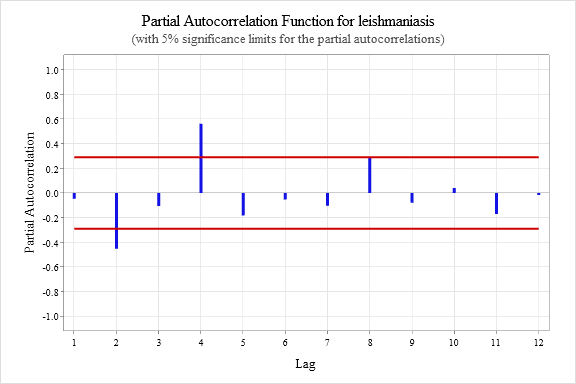


The shadow area is 95% confidence intervals.

**Supplementary figure 2- The distributions of the Leishmaniasis according to the cities and the years (from 2009-2019)**


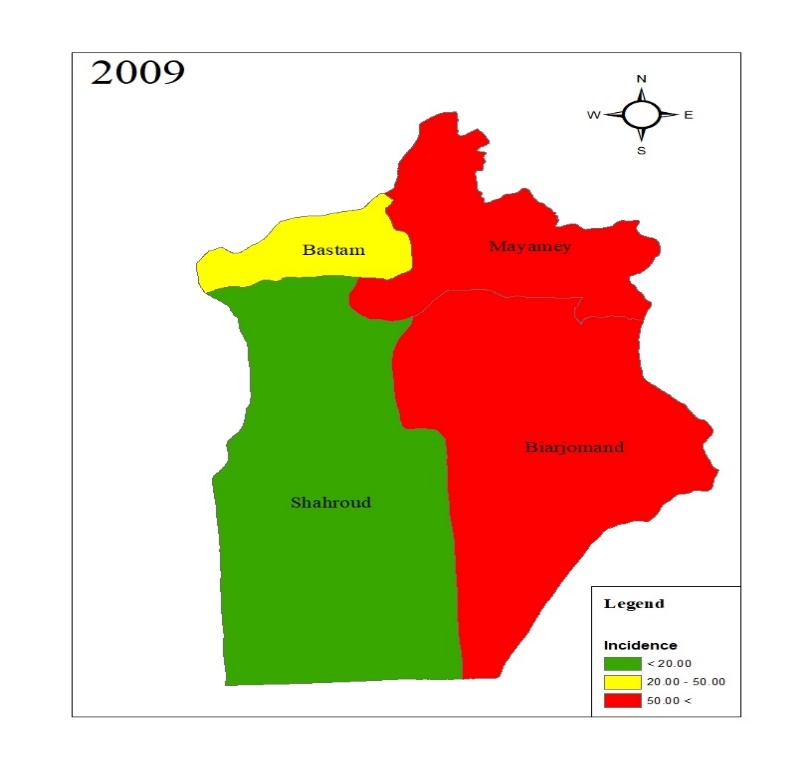


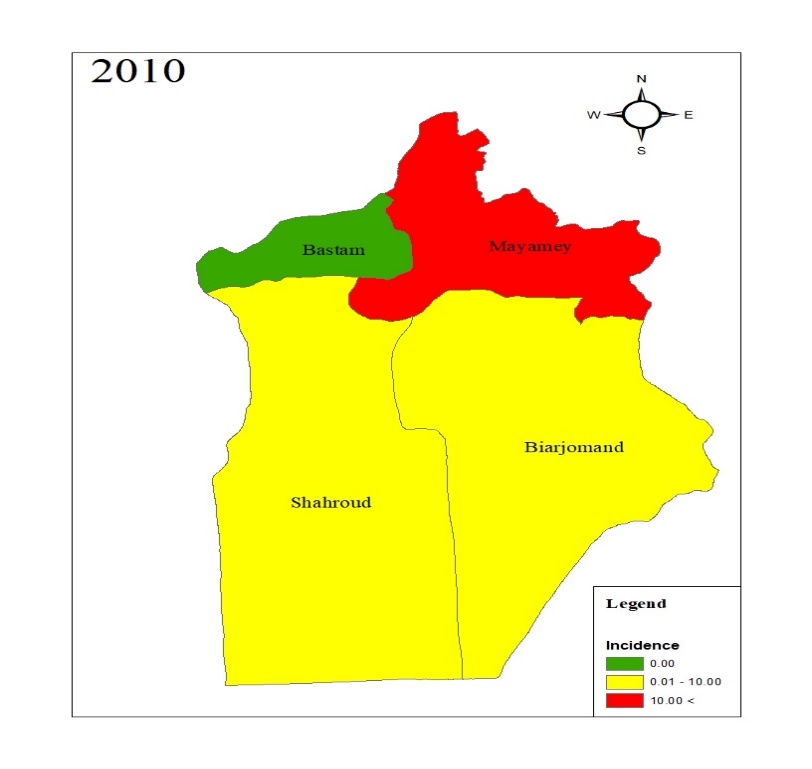


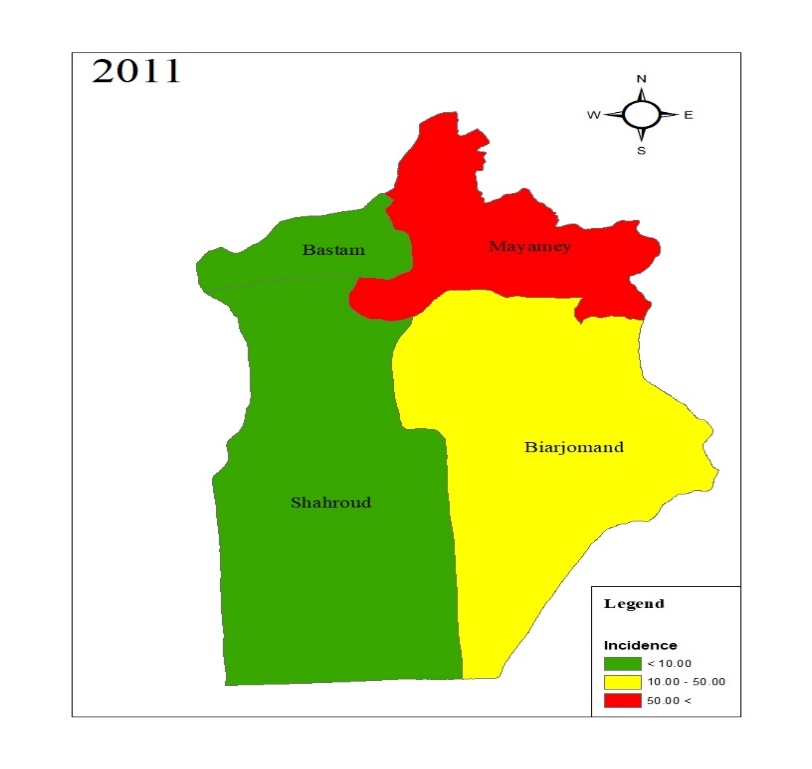

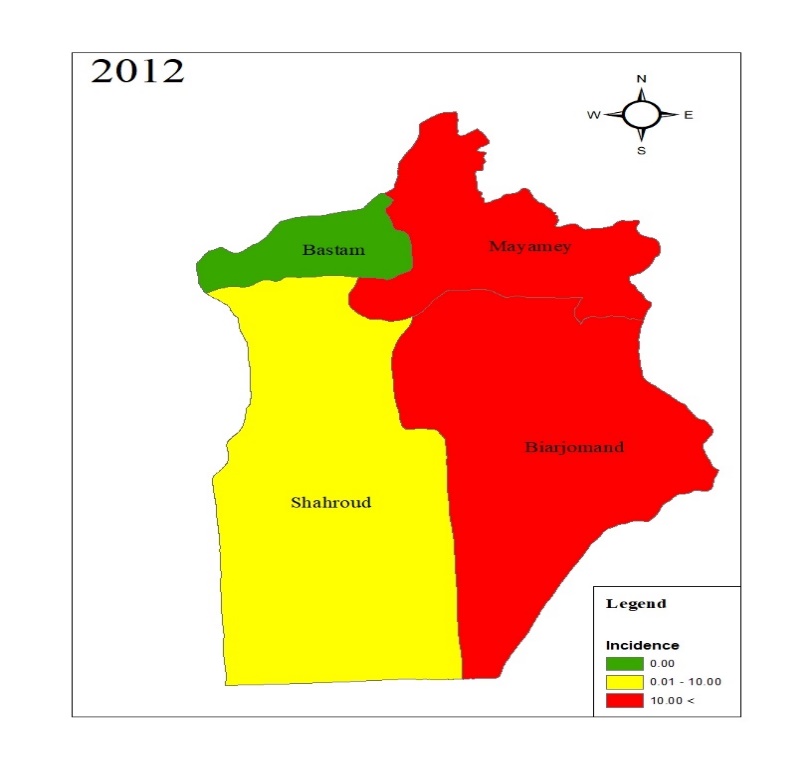


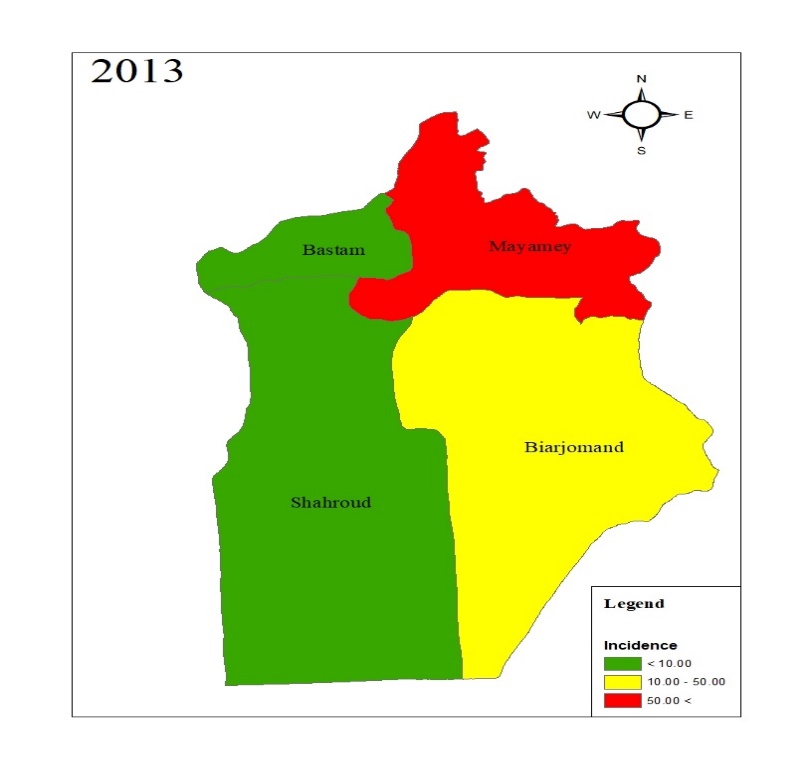

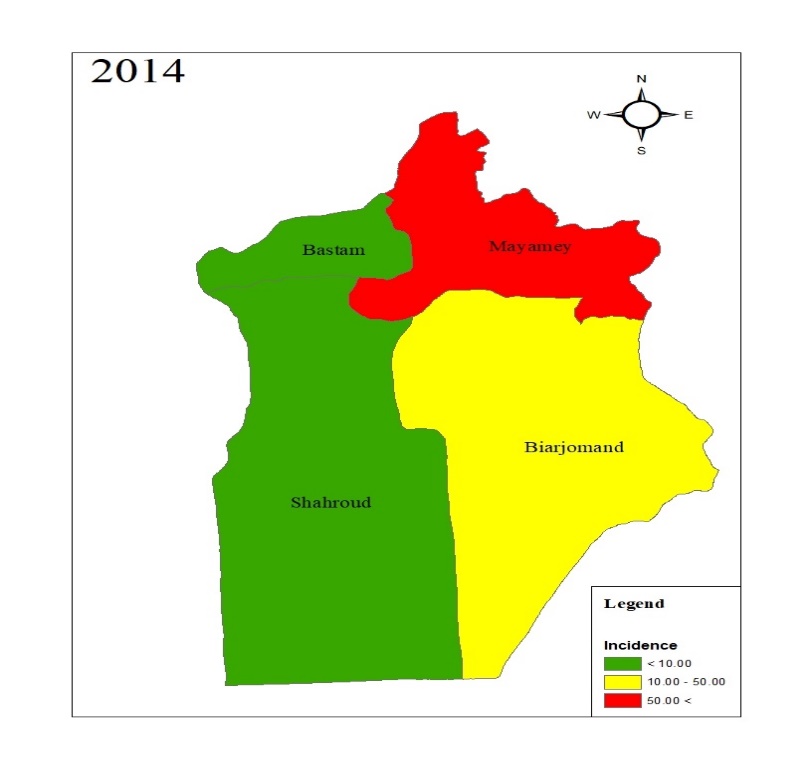


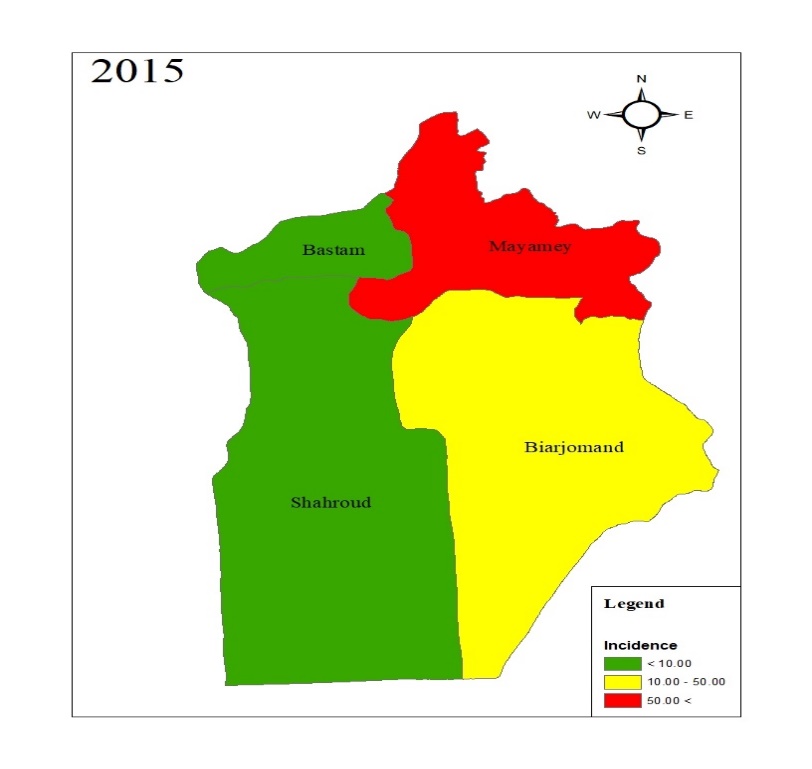

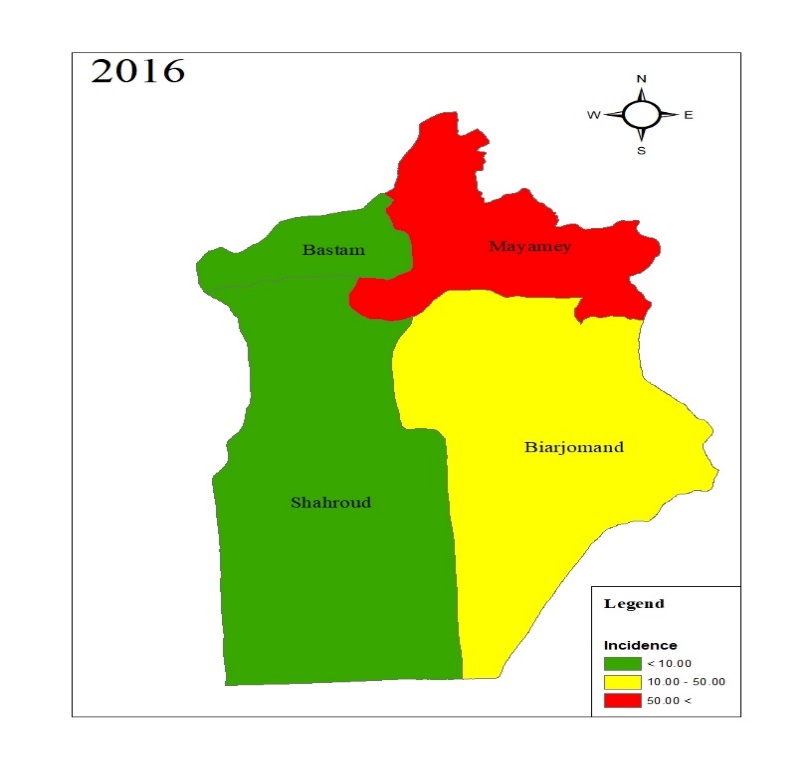


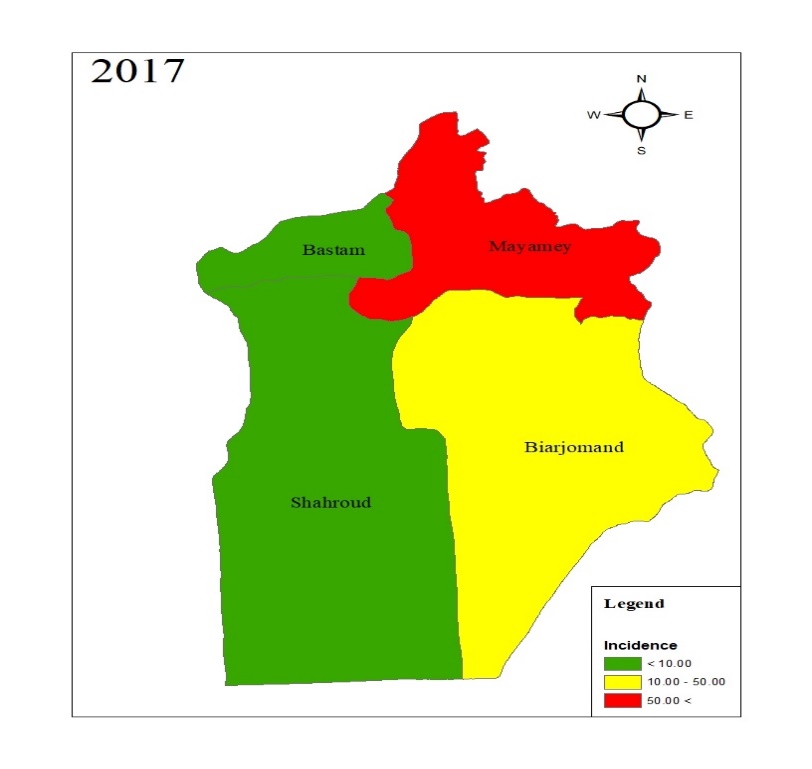

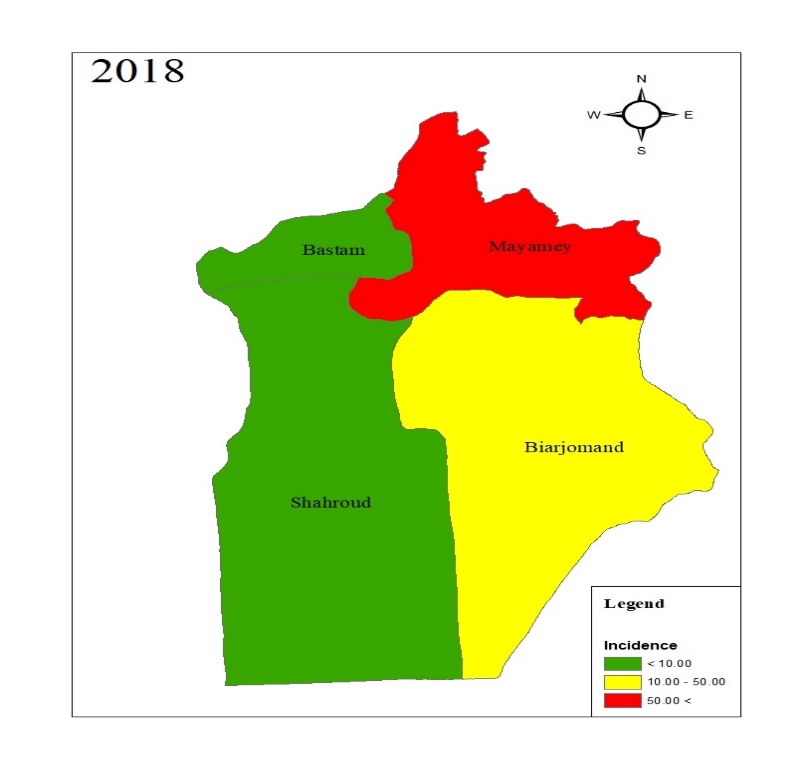


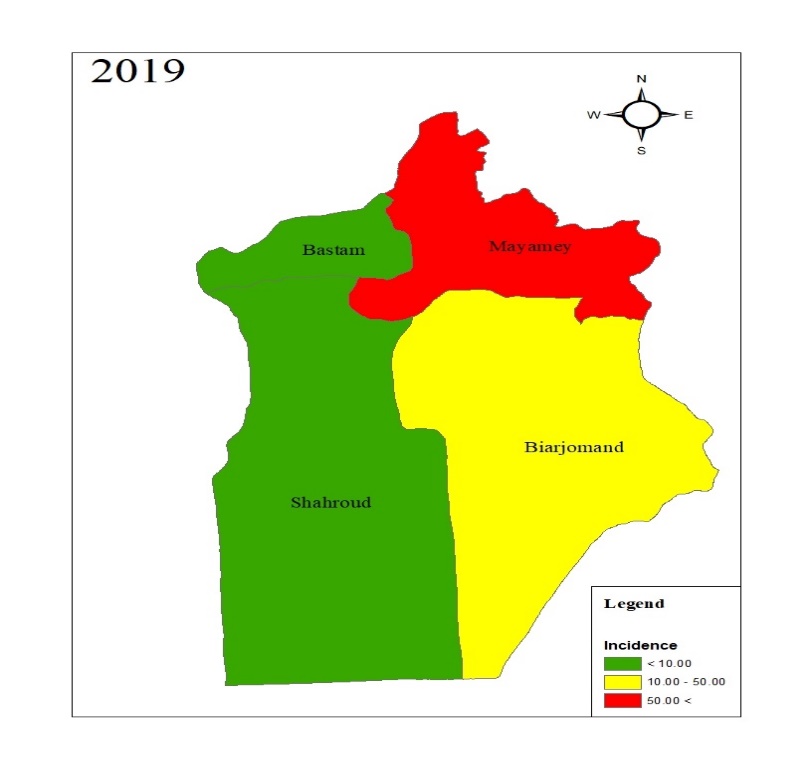

Supplement: Supplementary file 1 — Supplementary Material 1 [file 12889_2023_16121_MOESM1_ESM.docx]
